# Muscle-selective RUNX3 dependence of sensorimotor circuit development

**DOI:** 10.1242/dev.181750

**Published:** 2019-10-15

**Authors:** Yiqiao Wang, Haohao Wu, Pavel Zelenin, Paula Fontanet, Simone Wanderoy, Charles Petitpré, Glenda Comai, Carmelo Bellardita, Yongtao Xue-Franzén, Rosa-Eva Huettl, Andrea B. Huber, Shahragim Tajbakhsh, Ole Kiehn, Patrik Ernfors, Tatiana G. Deliagina, François Lallemend, Saida Hadjab

**Affiliations:** 1 Department of Neuroscience, Karolinska Institutet, Stockholm 17177, Sweden; 2 Department of Developmental and Stem Cell Biology, Institut Pasteur, CNRS UMR3738, Paris 75015, France; 3 Department of Neuroscience, University of Copenhagen, Copenhagen 2200, Denmark; 4 Helmholtz Zentrum München, German Research Center for Environmental Health, Institute of Developmental Genetics, Neuherberg 85764, Germany; 5 Unit of Molecular Neurobiology, Department of Medical Biochemistry and Biophysics, Karolinska Institutet, Stockholm 17177, Sweden; 6 Ming Wai Lau Centre for Reparative Medicine, Stockholm node, Karolinska Institutet, Stockholm 17177, Sweden

**Keywords:** Sensory system, Sensorimotor circuit, Dorsal root ganglia, Neuronal specification, Neurotrophins

## Abstract

The control of all our motor outputs requires constant monitoring by proprioceptive sensory neurons (PSNs) that convey continuous muscle sensory inputs to the spinal motor network. Yet the molecular programs that control the establishment of this sensorimotor circuit remain largely unknown. The transcription factor RUNX3 is essential for the early steps of PSNs differentiation, making it difficult to study its role during later aspects of PSNs specification. Here, we conditionally inactivate *Runx3* in PSNs after peripheral innervation and identify that RUNX3 is necessary for maintenance of cell identity of only a subgroup of PSNs, without discernable cell death. RUNX3 also controls the sensorimotor connection between PSNs and motor neurons at limb level, with muscle-by-muscle variable sensitivities to the loss of *Runx3* that correlate with levels of RUNX3 in PSNs. Finally, we find that muscles and neurotrophin 3 signaling are necessary for maintenance of RUNX3 expression in PSNs. Hence, a transcriptional regulator that is crucial for specifying a generic PSN type identity after neurogenesis is later regulated by target muscle-derived signals to contribute to the specialized aspects of the sensorimotor connection selectivity.

## Abstract (precis)

**Summary:** Spatial patterning of transcription factor activity among proprioceptive sensory neurons innervating different targets is required for their further specification and for sensorimotor connectivity.

## INTRODUCTION

The neuromuscular circuitry that controls all body movements relies on constant sensory feedback from the periphery to coordinate its commands to hundreds of muscles. The sensory components of this feedback are the proprioceptive sensory neurons (PSNs) of the dorsal root ganglia (DRG), which convey information from individual muscles to specific neuron groups in the spinal cord. Previous studies have indicated that the basic design of this sensorimotor circuit is already established at birth (Mears and Frank, 1997), and that its construction is largely independent of patterned neuronal activity (Frank, 1990; Mendelson and Frank, 1991; Mendelsohn et al., 2015), implying differential recruitment of specific molecular pathways during the establishment of the sensorimotor connections.

PSNs are identified by their specific co-expression of the runt related transcription factor RUNX3, tropomyosin receptor kinase C (TRKC, receptor for neurotrophin 3, NT3; also known as Ntrk3), the ETS transcription factor ER81, parvalbumin (PV) and the vesicular glutamate transporter 1 (VGLUT1) (Oliveira et al., 2003; Lallemend and Ernfors, 2012). Peripherally, they terminate in muscles and innervate the Golgi tendon organ (GTO) (Ib PSNs) and muscle spindles (MSs) (Ia and II PSNs). Centrally, their axons connect with distinct classes of interneurons and α-motor neurons (MNs) in the deep dorsal and the ventral horn of the spinal cord (Lallemend and Ernfors, 2012). Only Ia proprioceptive afferents make monosynaptic connections with MNs, establishing the sensorimotor reflex arc. This circuit is very selective as Ia afferents connect to MNs supplying the same muscles and avoid making connections with MNs commanding antagonistic muscles (Eccles et al., 1957; Frank and Mendelson, 1990). Most studies aimed at understanding the molecular programs that control the development of the motor circuits in relation to the anatomical identity of the peripheral connections have focused on the spinal MN specification (Dasen, 2009; Dasen et al., 2005; Price et al., 2002). Spinal MNs innervating the limbs are organized into distinct anatomical columns, the identity of which is tightly controlled by intrinsic genetic programs during early stages of MNs development, before they project peripherally into the limb, and which are essential for the construction of the stereotypic connections with individual muscles (Dasen, 2009; Dasen et al., 2005; Price et al., 2002; De Marco Garcia and Jessell, 2008). Unlike MNs, PSNs innervating a specific muscle are scattered in a mosaic fashion throughout multiple DRGs (Honig, 1982; Honig et al., 1998), and do not seem to possess any specific identity other than a generic expression of genes common to all PSNs before innervating their muscle target (Wu et al., 2019). PSNs instead would acquire subclass identities through extrinsic, presumably target-derived, signals days after their neurogenesis (Wu et al., 2019; Poliak et al., 2016). Hence, genetic manipulation of signaling pathways or transcription factors affecting the afferent outgrowth and muscle targeting of PSNs often results in a lack of sensorimotor connections (Lallemend et al., 2012; Levanon et al., 2002; De Nooij et al., 2013; Patel et al., 2003). For example, deletion of RUNX3, which drives the specification of PSNs and is associated with a complete absence of muscle proprioceptive innervation (Lallemend et al., 2012), results in a large deficit of central innervation (Nakamura et al., 2008). Similarly, without NT3-TRKC signaling, PSNs fail to innervate their peripheral muscle targets and their central projections do not extend further into the ventral horn of the spinal cord (Patel et al., 2003). Earlier studies in chick and a more recent study in mice provide strong support for a role of the target in assigning muscle-specific identities of PSNs that are likely to play a role in the establishment of specific patterns of central sensorimotor connections (Poliak et al., 2016; Wenner and Frank, 1995). However, with the exception of a role for Sema3E-PlexinD1 signaling in gating one specialized aspect of sensorimotor connectivity (Fukuhara et al., 2013; Pecho-Vrieseling et al., 2009), little is known about the molecular mechanisms required for the establishment of sensorimotor connections between selective PSNs and the central motor network. Here, using an experimental strategy that depletes RUNX3 expression after peripheral innervation, we uncovered a transcriptional link between peripheral muscle target, likely involving NT3 levels, and selective sensorimotor connectivity during late fetal stages.

## RESULTS

### RUNX3 is essential for the maintenance of cell identity of a subgroup of PSNs

**Fig. 1.**
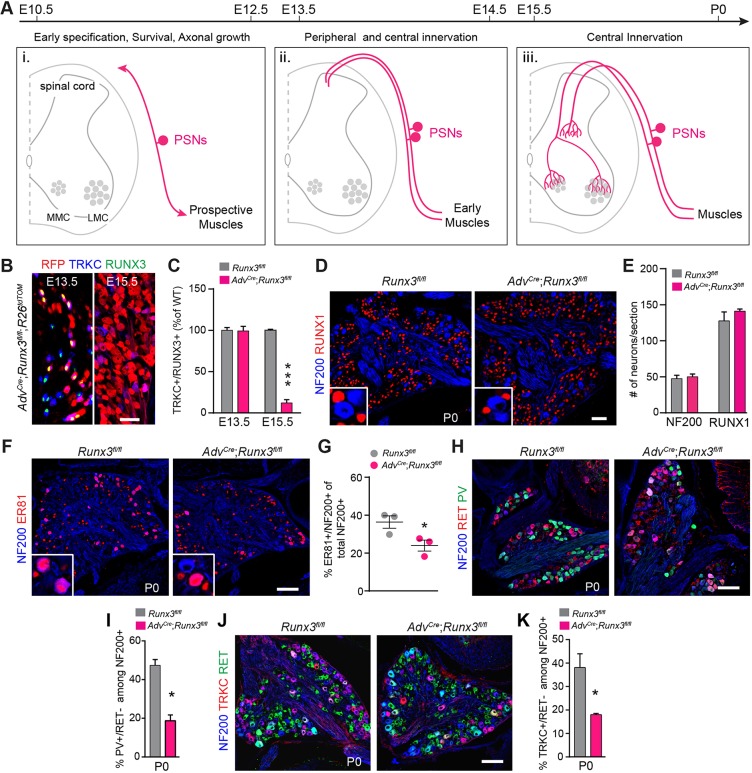
**Loss of cell identity in subgroups of PSNs following conditional targeting of RUNX3 after peripheral innervation.** (A) Scheme representing the successive developmental steps of PSNs, which contribute to sensorimotor circuit. Early specification of PSNs (i) is followed by peripheral axonal growth and muscle targeting (circa. E14) (ii). After peripheral innervation, central afferents of PSNs project to the intermediate and then ventral regions of the spinal cord to contact interneurons and motor neurons (iii). (B) Ablation of *Runx3* from sensory neurons using *Adv^Cre^* mice. At E13.5, RUNX3 expression is detectable in TRKC^+^ neurons with tdTomato (RFP) starting to be expressed in few neurons, while at E15.5, the recombination is fully efficient, all neurons expressing tdTomato and RUNX3 are strongly reduced in number. Scale bar: 50 µm. (C) Quantification of B, showing the recombination efficiency in TRKC^+^/RUNX3^+^ in *Adv^Cre^*;*Runx3^fl/fl^* mice (*n*=3). ***P≤0.001; Student's *t*-test. Data are mean±s.e.m. (D) Immunostaining for NF200 and RUNX1 on DRG sections from P0 *Adv^Cre^*;*Runx3^fl/fl^* and *Runx3^fl/fl^* animals identifies all myelinated sensory neurons (mechanoreceptive and proprioceptive neurons) and a large majority of nociceptive neurons (Lallemend and Ernfors, 2012; Gascon et al., 2010). Scale bar: 50 µm. (E) Quantification of D reveals absence of cell death in DRG neurons in the conditional *Runx3* mutants at P0. *P*>0.05, Student's *t*-test. Data are mean±s.e.m. (*n*=3). (F-K) Immunostaining for PSNs markers (F,H,J) and their quantification (G,I,K) in *Adv^Cre^*;*Runx3^fl/fl^* and *Runx3^fl/fl^* P0 animals (*n*=3 per genotype). Scale bars: 100 µm in F; 50 µm in H,J. *n*=3 per genotype; *P≤0.05; Student's *t*-test. (G) Data are mean±s.e.m.

To investigate the role of RUNX3 on the development of PSNs and of the sensorimotor circuits independently of its function on neuronal survival and early aspects of peripheral innervation – which end at E12.5 and E14.0, respectively (Fig. 1A) (Fariñas et al., 1998; Tourtellotte et al., 2001) – we first generated *PV^Cre^*;*Runx3^fl/fl^* mice, with *loxP*-flanked *Runx3* alleles and Cre expressed in PV-expressing neurons. The *PV^Cre^* mouse line induced recombination in a large majority of PV^+^ and RUNX3^+^ PSNs at E16.5 when analyzing brachial DRG from *PV^Cre^*;*R26^tdTOM^* mice (Fig. S1A,B). However, in *PV^Cre^*;*Runx3^fl/fl^* mice, RUNX3 deletion was observed only from birth, with a modest 20% reduction in RUNX3 expression at P0 (Fig. S1C,D), when the sensorimotor circuit is already established (Mears and Frank, 1997). We thus decided to generate *Adv^Cre^*;*Runx3^fl/fl^* mice, with Cre expressed under the control of the advillin gene (Zhou et al., 2010). Compared with the *PV^Cre^* driver line, the *Adv^Cre^* line induced recombination in all PSNs between E13.5 and E15.5, and a cross with *Runx3^fl/fl^* mice resulted in a near-complete absence of RUNX3 expression in DRG at E15.5 (Fig. 1B,C; Fig. S1E,F). Together, this confirms the use of *Adv^Cre^* to delete *Runx3* in PSNs just after their peripheral innervation, and at the time they are growing their axons centrally within the spinal cord to reach their target. In P0 *Adv^Cre^*;*Runx3^fl/fl^* mice, focusing our analysis on the brachial segments, the number of NF200^+^ DRG neurons (NF200 labels all myelinated DRG neurons, including PSNs) (Usoskin et al., 2015), and that of RUNX1^+^ DRG neurons, which represent a large proportion of the unmyelinated DRG neurons at birth (Lallemend and Ernfors, 2012; Gascon et al., 2010), were similar between mutants and control animals (Fig. 1D,E). Hence, the absence of RUNX3 after peripheral innervation does not affect neuronal survival at this stage. However, the expression of factors necessary for the proper development and function of PSNs, such as ER81 and TRKC, and of the marker PV were all downregulated in P0 *Adv^Cre^*;*Runx3^fl/fl^* DRG compared with *Runx3^fl/fl^* control mice (Fig. 1F-K). These results show that expression of RUNX3 in late embryonic DRG neurons is necessary for maintaining cell identity of a subgroup of PSNs.

### RUNX3 regulates development of central projection of subgroups of PSNs

**Fig. 2.**
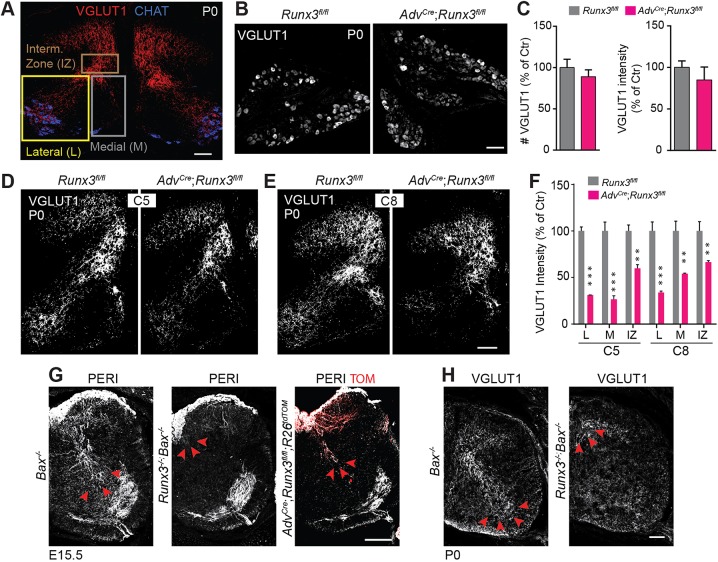
**Central afferentation deficit of PSNs after conditional targeting of RUNX3.** (A) Representative regions of the spinal cord analyzed. To reveal central afferent terminations of PSNs, we used VGLUT1 immunostaining on cross-sections of spinal cord. The pattern of VGLUT1 reactivity was analyzed in three reference regions: the intermediate zone (IZ), the ventromedial (M) and the ventrolateral (L) regions. Scale bar: 100 µm. (B) VGLUT1 expression in *Adv^Cre^*; *Runx3^fl/fl^* and *Runx3^fl/fl^* DRG sections at P0. Scale bar: 50 µm. (C) Quantification of the number of VGLUT1^+^ neurons per DRG section (left panel) and VGLUT1 intensity per cell (right panel) (from data in B) reveals absence of change in VGLUT1 expression in DRG from *Adv^Cre^*;*Runx3^fl/fl^* mice (*n*=3). (D,E) Central innervation of PSNs in *Adv^Cre^*;*Runx3^fl/fl^* and *Runx3^fl/fl^* mice at C5 (D) and C8 (E), as revealed by VGLUT1 immunostaining. Scale bar: 100 µm. (F) Quantification of the density of VGLUT1 staining in D and E in regions defined in A on one side of the spinal cord reveals deficits in central ingrowth of PSN afferents in conditional *Runx3* mutant mice (*n*=4 per genotype). A greater difference was observed in the lateral and medial (L and M) regions of the ventral spinal cord, which corresponds to the innervation of the MN pools (CHAT^+^ in A). **P≤0.01, ***P≤0.001; Student's *t*-test. Data are mean±s.e.m. (*n*=3). (G) Immunostaining for peripherin (PERI) on spinal cord sections shows complete absence of central PSN afferents in *Runx3^−/−^*;*Bax^−/−^* compared with *Adv^Cre^*;*Runx3^fl/fl^* mice (see red arrowheads). Scale bar: 100 µm. (H) Similar to G, at P0 VGLUT1 immunostaining on spinal cord sections confirms the absence of central axon growth of PSNs in *Runx3^−/−^*;*Bax^−/−^* mice (see red arrowheads), a phenotype that differs from *Adv^Cre^*;*Runx3^fl/fl^* mice (see D,E). Scale bar: 100 µm.

From E13.5 to E15.5, the central afferents of PSNs enter the dorsal part of the spinal cord and send axons toward the MNs, which they make contact with at ∼E17.5 (Mears and Frank, 1997; Ozaki and Snider, 1997). The development of the proprioceptive axonal inputs in the spinal cord has been suggested to involve NT3 and the transcription factors ER81 and RUNX3, with the deletion of any of them leading to severe deficits in the central projection pattern of PSNs (Patel et al., 2003; Nakamura et al., 2008; Arber et al., 2000). Yet those three factors are necessary for the peripheral outgrowth of PSNs and for their survival (De Nooij et al., 2013; Lallemend et al., 2012; Patel et al., 2003), preventing any study of their direct function on sensorimotor connectivity using null mutant mice. Here, we have explored whether the loss of RUNX3 in PSNs from E15 could perturb the formation of the sensorimotor connections between PSNs and MNs of the ventral spinal cord (phase iii, Fig. 1A). To this end, we analyzed the distribution of VGLUT1^+^ sensory bouton contacts with CHAT^+^ (choline acetyl transferase) MNs at P0, which reflects direct excitatory inputs from Ia PSNs on MNs (Oliveira et al., 2003; Pecho-Vrieseling et al., 2009). The innervation territory of PSNs in the spinal cord was divided into three distinct compartments (Fig. 2A): a ventro-medial (M) and a ventro-lateral (L) region corresponding presumably to axial and hypaxial muscle-derived Ia projections, respectively; and an intermediate zone (IZ), where most GTOs and type II MS afferents project (De Nooij et al., 2013; Lallemend and Ernfors, 2012). In *Adv^Cre^*;*Runx3^fl/fl^* and *Runx3^fl/fl^* mutant pups, VGLUT1 expression levels appeared unchanged in PSNs cell bodies (Fig. 2B,C). We observed a general decrease in the density of VGLUT1 labeling in these three regions at all brachial levels in the mutant (here shown for C5 and C8, Fig. 2D-F), with the largest defect found in the lateral and medial part of the ventral horn, which corresponds to the innervation territory of MNs (scheme in Fig. 1A; Fig. 2F). However, no defect was visible at the thoracic level (Fig. S2A). These results contrasted with the phenotype observed in the *Runx3^−/−^*; *Bax^−/−^* embryos in which, in spite of the absence of sensory neuron cell death due to null mutation of the pro-apototic gene *Bax* (Deckwerth et al., 1996), their central afferents did not grow beyond the dorsal aspect of the spinal cord, as revealed by immunostaining for peripherin at E15.5 and VGLUT1 at E18.5 (Fig. 2G,H). Taken together, our results indicate that PSNs at limb levels require RUNX3 expression to project correctly into the ventral spinal cord, independently of its role on the outgrowth dynamic of their peripheral projections. Nevertheless, some proprioceptive afferents still terminate both at the intermediate spinal cord and in the MN area in the absence of RUNX3. We thus hypothesized that RUNX3 might regulate the central connectivity of selective PSNs.

### Development of muscle-selective sensorimotor connections through RUNX3 activities

**Fig. 3.**
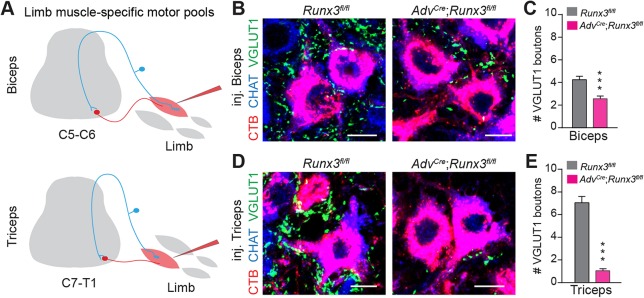
**Muscle-selective differential penetrance of central deficits of PSN connectivity in RUNX3 conditional mutants.** (A) Experimental scheme describing muscle injection of the antagonistic muscles biceps and triceps, and retrograde labeling of specific MN pools at spinal segments C5-C6 for the biceps or segments C7-T1 for the triceps. (B) Immunostaining of spinal cord cross-sections representing MNs (CHAT^+^, in blue) traced by the CTB (red) from initial injection in the biceps. PSN synaptic contacts with the MNs are visualized by the VGLUT1^+^ synaptic bouton (green). Scale bars: 10 µm. (C) Quantification of 38 MNs (from B) in *Runx3^fl/fl^* and 39 MNs in *Adv^Cre^*;*Runx3^fl/fl^*. ***P≤0.001; Student's *t*-test. Data are mean±s.e.m. (*n*=3). (D) Immunostaining of spinal cord cross-sections representing MNs (CHAT^+^, in blue) traced by the CTB (red) from initial injection in the triceps. PSN synaptic contacts with the MNs are visualized by the VGLUT1^+^ synaptic bouton (green). Scale bars: 10 µm. (E) Quantification of a total of 44 MNs (from D) in *Runx3^fl/fl^* and 46 MNs in *Adv^Cre^*;*Runx3^fl/fl^*. ***P≤0.001; Student's *t*-test. Data are mean±s.e.m. (*n*=3).

PSNs innervating specific skeletal muscles project centrally to and make contact with defined pools of MNs innervating the same muscles (Mears and Frank, 1997; Eccles et al., 1957; Dasen, 2009; Surmeli et al., 2011). This connection rule forms the structural basis of the muscle stretch reflex circuit. To explore whether the absence of RUNX3 affects preferentially the innervation of specific MN pools, we used Cholera toxin B subunit (CTB) injection into two major antagonistic groups of forelimb muscles: the biceps brachii (flexor muscles) and the triceps brachii (extensor muscles, Fig. 3A). Biceps and triceps CHAT^+^ MNs (MNs^Biceps^ and MNs^Triceps^, respectively) were retrogradely traced by injecting CTB in their respective muscles at postnatal day (P) 1, and their proprioceptive afferent terminals were examined at P2 by quantifying their number of contacts with VGLUT1^+^ boutons (Pecho-Vrieseling et al., 2009; Surmeli et al., 2011). The majority of MNs^Biceps^ were found essentially in C5-6 spinal segments, while MNs^Triceps^ were found in C7-T1 spinal segments, as previously shown (Vrieseling and Arber, 2006). Gamma MNs, identified by their small size, were not considered for analysis. Quantification of the number of VGLUT1^+^ boutons making synapses with MNs^Biceps^ revealed a 40% decrease in *Adv^Cre^*;*Runx3^fl/fl^* mice compared with *Runx3^fl/fl^* control littermates (Fig. 3B,C). Strikingly, the majority of VGLUT1^+^ boutons synapsing onto MNs^Triceps^ were lost in the mutant (Fig. 3D,E), suggesting that RUNX3 expression in late embryonic stage has a selective role in the formation of sensorimotor connections between PSNs and defined pools of MNs.

### Selective loss of the peripheral innervation of PSNs in the absence of RUNX3

**Fig. 4.**
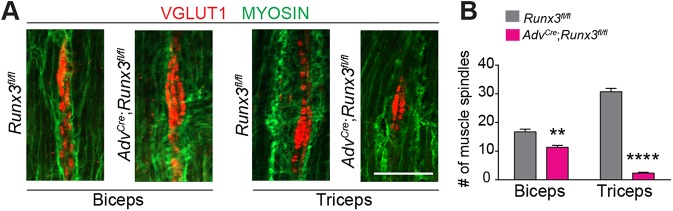
**Muscle-specific MSs deficits in conditional *Runx3* mutants at birth.** (A) Immunostaining for VGLUT1 (for MSs) and myosin on cross-sections from biceps and triceps. (B) Quantification reveals a muscle-selective MS deficiency in *Adv^Cre^*;*Runx3^fl/fl^* with a significant decrease in the numbers of MSs in triceps (*n*=3 animals). **P≤0.01, ****P≤0.0001; Student's *t*-test. Data are mean±s.e.m. Scale bar: 100 µm.

We next wondered whether the preferential sensorimotor connection deficits observed in *Adv^Cre^*;*Runx3^fl/fl^* mice are reflected in the peripheral targeting of limb muscles by PSNs. MSs form after interaction between the sensory nerve endings of the PSNs and the targeted muscle fibers at ∼E14 (Tourtellotte et al., 2001). Here, we quantified at P0 the VGLUT1^+^ sensory endings associated with MSs as readout of muscle innervation by PSNs (Fig. 4A) (Poliak et al., 2016). The GTO-associated nerve endings of the PSNs were not considered in the analysis. We focused on two pairs of antagonistic muscles spanning the proximo-distal axis of the forelimb: the extensors triceps and extensor carpi radialis (ECR); and the flexors biceps and flexor carpi radialis (FCR). Strikingly, while the biceps and FCR muscles exhibited, respectively, a 30% decrease and normal incidence of VGLUT1^+^ PSN nerve endings in *Adv^Cre^*;*Runx3^fl/fl^* mice, the triceps and ECR muscles displayed a large reduction in PSN nerve endings (Fig. 4B; Fig. S3A,B). In addition, the few remaining MSs observed in triceps and ECR muscles of *Adv^Cre^*;*Runx3^fl/fl^* mutants were systematically of smaller size (Fig. 4A). These analyses at the forelimb level revealed a specific role for RUNX3 in PSN specification that affects MS differentiation in some defined muscle groups. They also show that the loss of intraspinal PSN axons targeting specific pools of MNs correlates well with the lack of sensory nerve endings in the limb muscles they innervate.

The observation that this innervation deficit is not associated with sensory neuron cell death in the *Adv^Cre^*;*Runx3^fl/fl^* mutants at P0 raises the issue of what happens to those particular PSNs that have lost contact with their peripheral target. The peripheral projections of brachial PSNs make dorso-ventral choice within the forelimb before E12.5 (Kania et al., 2000; Huettl et al., 2011), and reach their peripheral target at E14 (Tourtellotte et al., 2001). By deleting RUNX3 after this period using the *Adv^Cre^* driver, it is thus unlikely that the PSNs losing their synaptic connection and identity will retract their projections to target another opposite muscle or different sensory organ such as the skin. Indeed, both the intrinsic signaling and environmental cues might not enable these cells to adopt a different sensory cell fate or trajectory. However, although future studies will be necessary to resolve this issue, the observed loss of identity features could eventually lead to cell death at later stage, as shown after the loss of many terminal selector genes that are needed to maintain a differentiated state (Deneris and Hobert, 2014).

### Role of muscle target and NT3 signaling in sustained RUNX3 expression

**Fig. 5.**
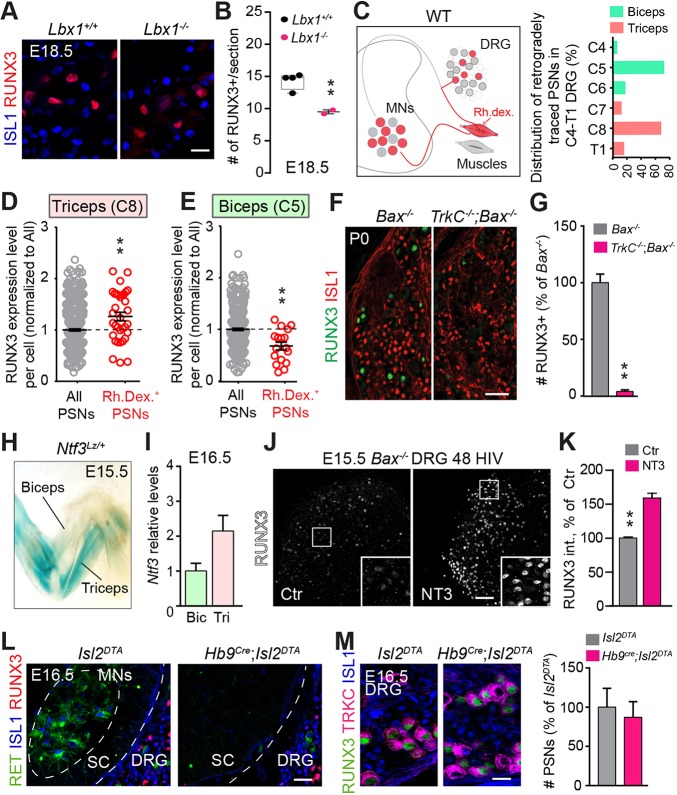
**RUNX3 dependence of PSN connectivity correlates with RUNX3 levels and muscle target NT3 levels.** (A) Immunostaining for ISL1 and RUNX3 on DRG sections from *Lbx1^−/−^* and *Lbx1^+/+^* mice. Scale bar: 50 µm. (B) Quantification of RUNX3^+^ neurons (from A) in *Lbx1^−/−^* (*n*=2) and their control littermates (*n*=4) at brachial levels shows significant reduction in the full mutants compared with control animals. **P≤0.01. Data are mean±s.e.m. (C) Experimental design. (Left) Rhodamine dextran (Rh. dex.) injection in specific muscle will retrogradely trace their innervating PSNs in the DRG from E16.5 wild-type animals. The dextran is injected in biceps in one of the forelimbs and in triceps in the contralateral limb. (Right) Distribution within DRG of the PSNs innervating biceps and triceps, showing biceps- and triceps-innervating PSNs located mostly in DRG C5 and C8, respectively (data from 5 animals for triceps and 11 animals for biceps). (D,E) Quantification of RUNX3 expression per cell in retrogradely traced PSNs (as in C) versus all RUNX3^+^ PSNs after injection of Rh. dex. in triceps (analysis at C8 level, D) or in biceps (analysis at C5 level, E). **P≤0.01; Student's *t*-test. Data are mean±s.e.m. (single neurons analyzed from five embryos). (F) RUNX3 expression is largely reduced in *TrkC^−/−^*;*Bax^−/−^* P0 mice: a mouse model of peripheral outgrowth deficits. Scale bar: 100 µm. (G) Quantification of F reveals an almost complete absence of RUNX3 expression in *TrkC^−/−^*;*Bax^−/−^* P0 mice compared with their control littermates (*n*=2). **P≤0.01; Student's *t*-test. Data are mean±s.e.m. (H) X-Gal reaction on E15.5 in *Nft3^Lacz/+^* forelimb embryos show heterogeneous muscle-specific expression of NT3. There is a large difference in NT3 levels between biceps and triceps. (I) Quantification of NT3 mRNA (*Nft3*) in the biceps (Bic) and triceps (Tri) shows a twofold increase in triceps compared with biceps in control animals (Ctr). *P*=0.052; Student's *t*-test (*n*=2 samples from four animals). Data are mean±s.e.m. (J) *Bax^−/−^* mice DRG explants (E15.5) in culture with or without NT3 for 48 h *in vitro* (HIV) reveal a decreased expression of RUNX3 in the absence of NT3. Scale bar: 100 µm. (K) Quantification of J shows a significant increase of RUNX3 intensity per cell, while the number of positive neurons remains unchanged. **P≤0.01; Student's *t*-test. Data are mean±s.e.m. (*n*=3). (L) Immunostaining for ISL1, RET and RUNX3 on spinal cord (SC) sections from *Hb9^Cre^*;*Islet2^DTA^* mice shows complete absence of MNs at E16.5. Scale bar: 50 µm. (M) Immunostaining for TRKC, RUNX3 and ISL1 on DRG sections from *Hb9^Cre^*;*Islet2^DTA^* and *Islet2^DTA^* E16.5 mice shows no deficits in RUNX3 and TRKC expression in the absence of MNs, confirmed by the quantification of the number of PSNs in *Hb9^Cre^*;*Islet2^DTA^* and *Islet2^DTA^* (*n*=2, right panel). Scale bar: 20 µm.

We next asked whether the phenotypic defects observed in PSNs in our *Adv^Cre^*;*Runx3^fl/fl^* mutants could reflect a role for muscle-derived signals in PSN afferent specification. To assess this, we analyzed RUNX3 expression in *Lbx1* null mice, in which all extensor muscles are lacking at forelimb level although a large number of flexor muscles still develop (Gross et al., 2000; Brohmann et al., 2000). However, this phenotype is not accompanied by a loss in PSN number or by a defect in the peripheral outgrowth of PSN afferents within the limbs (Poliak et al., 2016). Examining brachial DRG neurons from E18.5 *Lbx1^−/−^* mice, we observed a 40% reduction in the number of neurons expressing RUNX3 (Fig. 5A,B). Interestingly, the remaining RUNX3^+^ cells in *Lbx1* mutants exhibited reduced levels of expression similar to low RUNX3-expressing cells in wild-type animals [*Lbx1^+/+^*, 1±0.077; *Lbx1^−/−^*, 0.401±0.069 (expressed as mean±s.d.); *P*<0.01, normalized to *Lbx1^+/+^*], indicating that RUNX3 levels in PSNs might correlate with the specific target muscle groups they innervate. To test this, we analyzed backfilled PSNs following retrograde tracing using rhodamine dextran (Rh.dex.) injection in triceps and biceps from E16.5 wild-type embryos (Fig. 5C). Biceps- and triceps-innervating PSNs were primarily found in C5 and C8 segments, and expressed (on average) low and high levels of RUNX3, respectively, when compared with the whole PSN population (Fig. 5C-E; Fig. S4A). Together, these data suggest that RUNX3 expression and its level in PSNs depend on limb muscles at late embryonic stages. To support this, we analyzed *Bax^−/−^* mice devoid of NT3 signaling and in which PSNs completely fail to project into limb muscles but survive even in reduced neurotrophic factor conditions (Patel et al., 2003). RUNX3 expression was dramatically decreased in E15.5 *TrkC^−/−^*; *Bax^−/−^* DRG and virtually absent at birth (P0; Fig. 5F,G; Fig. S4B). Its expression in E13.5 *TrkC^−/−^*;*Bax^−/−^* DRG, i.e. prior to target innervation, however, was unchanged (Fig. S4B), indicating a late function of muscle-derived signals, following sensory afferent-muscle fiber contact, in the regulation of RUNX3 expression in PSNs. NT3 itself was shown to be a plausible candidate for controlling later aspects of PSNs specification (Wang et al., 2007; De Nooij et al., 2013). Notably, elevated levels of NT3 in muscles late during embryonic development has been shown to disrupt the selective pattern of synaptic connections between sensory afferents and MNs (Wang et al., 2007). In addition, various levels of NT3 in hindlimb muscles have been correlated with subclass-specific sensitivity of PSNs to cell death following loss of ER81 (De Nooij et al., 2013). This heterogeneity in NT3 levels among muscles was also observed here in forelimb muscles using β-gal activity in whole-mounted *Ntf3*^*LacZ*/+^ E15.5 embryos and confirmed using qPCR (Fig. 5H,I). To directly test the role of NT3 on RUNX3 expression at late embryonic stage, we cultured E15.5 DRG explants from *Bax^−/−^* embryos for 2 days in the presence or absence of NT3 (Fig. 5J). Sensory neurons from *Bax^−/−^* DRG could survive several days *in vitro* without neurotrophin signaling (Lentz et al., 1999; Zhong et al., 2007). We found higher levels of RUNX3 expression in the presence of NT3 compared with the control condition (Fig. 5K). Supporting these data, addition of NT3 to DRG explants from HH27 chicken embryos resulted in marked upregulation of *Runx3* mRNA levels compared with untreated DRG explants (Fig. S4C,D). Thus, after peripheral innervation, distinct levels of RUNX3 expression in PSN subgroups are controlled by muscle-derived signals and involve NT3 signaling.

We also considered whether MNs, through direct axon-axon contact or indirect signaling, could act on RUNX3 expression in PSNs. To test this, we used *Hb9^cre^*;*Isl2^DTA^* mice, in which diphtheria toxin (DTA) is selectively expressed in HB9^+^/ISL2^+^ MNs, ablating MNs as they exit the cell cycle, before peripheral innervation (Yang et al., 2001) (Fig. 5L). In the absence of MNs, PSNs density in DRG is unchanged, and their nerve endings can still be found in association with MSs (Poliak et al., 2016). In *Hb9^cre^*; *Isl2^DTA^* mice examined at E16.5, RUNX3 expression in PSNs was found unchanged compared with *Isl2^DTA^* mice (Fig. 5M). These data thus argue against the idea that MNs could provide signals necessary for regulating or maintaining RUNX3 in PSNs at late embryonic stages.

## DISCUSSION

Muscle sensory feedback is essential for controlling coordination of locomotor behavior. It is conveyed by different subgroups of PSNs that form specific connections with second order neurons and MNs in the spinal cord. Despite the importance of this feedback in motor behavior, our knowledge of the molecular programs that control the assembly of the sensorimotor circuits remains limited. This is essentially due to the fact that the major genetic determinants of PSNs identity, which continue to be expressed in the mature neurons, are also necessary for their early peripheral outgrowth and survival, limiting the study of their direct function in stage-specific maintenance of PSNs identity and connectivity. Here, we have used conditional Cre/*loxP* gene-targeting approach to delete the major regulatory factor *Runx3* in PSNs after the period of natural cell death and peripheral innervation. We find that RUNX3 is continuously required throughout development to maintain specific identity features of particular subgroups of PSNs innervating the limb, independent of cell death. We also show that the maintained expression of RUNX3 in PSNs is regulated by muscle target-derived signals and NT3 signaling which is differentially required in subgroups of PSNs to setup specific sensorimotor connections.

### Dedicated maintenance factors for PSN subgroups

The generation of proprioceptive afferents during embryonic development follows a systematic sequence of events, from neurogenesis, early cell fate specification and target innervation to finer aspects of PSN subgroup-specific identity and the formation of the sensorimotor circuits (Lallemend and Ernfors, 2012). Hence, soon after neurogenesis, TRKC^+^ PSNs acquire RUNX3 expression, which is necessary to consolidate a proprioceptive fate and direct the extent of peripheral outgrowth of PSNs projections (Lallemend et al., 2012; Levanon et al., 2002; Kramer et al., 2006). As their axons grow peripherally, PSNs, through target-derived NT3 signaling, will initiate expression of ER81, which later will define the survival competence of particular subgroups of PSNs in correlation with their target muscles (Patel et al., 2003; De Nooij et al., 2013). At the same time, different subtypes of PSNs emerge on the basis of their preferential expression of recognition molecules defined by interactions with signals notably from the limb (Poliak et al., 2016; Wu et al., 2019). In this context, it has been unclear whether early specification markers continue to be crucial during late embryogenesis for preservation and maturation of the sensory neuron type identity and connectivity. Our data indicate that RUNX3 is not essential for the survival of PSNs following peripheral innervation. This suggests that the loss of PSNs observed in *Runx3*-null mice at E12.5 is likely a consequence of the severe reduction in TRKC expression and survival signaling within PSNs during the developmental cell death period (Lallemend et al., 2012; Levanon et al., 2002; Kramer et al., 2006). This is reminiscent of a recent study exploring the role of BRN3A in the diversification of sensory neurons of the cochlea, where conditional deletion of *Pou4f1* (encoding BRN3A) from E14.5 (while it is first expressed at E10.5) did not affect the survival of cochlear neurons as seen in the full mutant where TRKC is found to be downregulated (Huang et al., 2001; Sherrill et al., 2019). In contrast, classic key molecular features of PSN identity, such as ER81, TRKC and PV expression, showed a deficit in our conditional *Runx3* mutant. Interestingly, only a subgroup of PSNs, representing about half of its population at the forelimb level, showed a loss of identity marker expression. This implies the existence of two distinct subgroups of PSNs at limb levels based on their dependence on RUNX3 expression for the later aspect of their differentiation. In support of this, key molecular markers of PSNs are found unchanged in thoracic DRG in our conditional *Runx3* mutant mice. One possible mechanism that could participate in the development of these two distinct subgroups of PSNs is the graded level of RUNX3 activity itself. A previous study in chicken embryos using *in ovo* electroporation suggested that the status of RUNX3 expression could participate in the segregation of the different subtypes of PSNs (innervating GTOs or MSs), notably by regulating their central projection trajectory (Chen et al., 2006). Our data in mice indicate instead that the levels of RUNX3 expression and its role in PSN diversification might correlate with the identity of the target muscles being innervated, with mean RUNX3 levels decreasing in remaining PSNs projecting to forelimb flexor muscles in *Lbx1* null mice, which are devoid of extensor muscles (Gross et al., 2000; Brohmann et al., 2000). These contrasting data could indicate a species difference. In support, siRNA-based extinction of RUNX3 expression in embryonic chicken PSNs did not lead to loss of TRKC (Chen et al., 2006), which would be expected from the data found in mice (Lallemend et al., 2012; Kramer et al., 2006). Our data remind us, however, of the differential activity of the NT3-ER81 signaling pathway in PSNs and its role in delineating two classes of PSNs based on their dependence on ER81 for their survival (De Nooij et al., 2013). Although results from our work would need further investigation of a direct link between RUNX3 levels and PSN diversification, graded levels of transcription factor activity may play a crucial role in the development of neuronal identity. In this context, the regional differences in the expression at the target of signaling cues might serve at the nerve endings as key factors eliciting various strengths of retrograde signaling operating in the neurons to drive their identity. A very good example is the development of the trigeminal ganglion, in which the coincident expression of both BDNF and BMP4 in discrete regions of the peripheral target field plays an important role in controlling the development of specific neuronal pools within the ganglion (Ji and Jaffrey, 2012). Distinct signals derived from limb mesenchyme have also been hypothesized to drive the subclass identity of PSNs. In a similar way, we show here that the presence of muscles is necessary for maintaining the expression of RUNX3 in PSNs. Moreover, NT3, which is expressed at various levels in developing skeletal muscles (Fariñas et al., 1996; De Nooij et al., 2013), can directly regulate RUNX3 expression *ex vivo*. Hence, it is conceivable that various extents of NT3-TRKC signaling activities between PSNs subgroups innervating distinct muscles might regulate their later cell identity aspects through graded RUNX3 expression. Following peripheral innervation, such differential activities among distinct PSNs subgroups could eventually serve to link the targeted muscles with the central neurons in the spinal cord the PSNs must connect.

### Selectivity of sensorimotor connectivity

Certainly the most studied circuit in sensorimotor network connecting PSNs and spinal cord neurons is the reflex arc in which group Ia PSN afferents make specifically strong connections with MNs supplying the same muscle and weaker connections with motor neurons supplying synergistic muscles, while no connection is established with MNs of antagonistic muscles (Eccles et al., 1957; Frank and Mendelson, 1990). During the formation of this circuit, an elegant study has shown that the topography of the PSN endings along the proximodistal axis of the limb could predict the dorsoventral location and identity of their target MNs, suggesting an area-specific targeting of PSNs within the ventral spinal cord (Surmeli et al., 2011). Others emphasized the importance of a direct MN-dependent mechanism that involves cell-to-cell recognition with PSNs and the use of Sema3E-plexin D1 signaling for the formation of sensorimotor connectivity patterns (Fukuhara et al., 2013; Pecho-Vrieseling et al., 2009). This implies a stepwise targeting approach making use of different signaling and strategies that PSN must use to eventually synapse to the appropriate MNs. Our data here on the selective function of RUNX3 in the spinal cord ingrowth of particular subgroups of PSNs indicate another level of regulation by which only a subset of neurons critically depends on the activity of a specific transcriptional program to project within the spinal cord and eventually develop a proper connectivity. Interestingly, this RUNX3 dependence of PSN-MN connections exhibits a preference for neurons innervating forelimb extensors, when compared with flexors and axial muscles at limb level, as well as thoracic muscles. In adult animals, such a deficit of muscle spindles in limb extensors, in acute situation, would most likely cause a substantial decrease in muscle tone of extensors, resulting in a change in the basic body configuration (a configuration with more flexed limbs as compared with control) and a decrease in efficacy of feedback postural corrections. In our mutant mice, due to adaptive plastic changes that could take place during early life, these deficits could be compensated for, e.g. by an increase in the activity of neurons of the vestibulospinal tract leading to an increase in extensor muscle tone and/or by a substitution of proprioceptive information (which in control subjects plays a crucial role in the generation of postural corrections) by visual and vestibular signals. However, the most likely specific aspects of postural corrections might still differ from those observed in control animals.

The RUNX3-dependent bias to neurons innervating forelimb extensors, compared with flexors muscles, evokes the ER81 dependence of PSNs for their survival, where PSNs innervating thoraco-hypaxial and axial muscles exhibit an almost complete dependence on ER81, whereas those innervating hindlimb muscles exhibit a mosaic, muscle-by-muscle, sensitivity to ER81 inactivation (De Nooij et al., 2013). Strikingly, the levels of NT3 expression in individual muscles strongly correlated with this dependence on ER81 of the survival of particular PSNs subgroups. Although our study only considers selected sensorimotor connections, it suggests a role for peripheral gradation of NT3 in setting up the sensitivity of PSNs for RUNX3-dependent sensorimotor connectivity, as described above. Even if further programs are certainly required to create the fine topography of PSNs to MNs connectivity, this earlier step already distinguishes populations of neurons as they need to enter the spinal cord, as shown in chick and mouse for the selectivity of the sensorimotor circuits by regionally restricted limb signals (Wenner and Frank, 1995; Poliak et al., 2016). This could serve to coordinate spatially and temporally separated developmental events, as observed during the development of olfactory receptor neurons and their selective connectivity with their glomerular target (Chou et al., 2010). The implication of these results is also that, within proprioceptors, higher levels of RUNX3 might engage a distinct set of transcriptional targets that are necessary for their proper maturation. The lower RUNX3-expressing PSNs would then require the activation of another set of transcriptional regulators. Such a mechanism, which most likely involves differences in target-derived signals, including neurotrophins, would thus participate in a spatial patterning of transcription factor activities among sensory neurons.

## MATERIAL AND METHODS

### Animals

Wild-type C57BL6 mice were used unless specified otherwise. *Runx3^−/−^*, *Advillin^Cre^* (*Adv^Cre^*) *Lbx1^−/−^*, *Hb9^Cre^* and *Isl2^DTA^* mouse strains have been described elsewhere (Levanon et al., 2002; Yang et al., 2001; Gross et al., 2000; Zhou et al., 2010). *Runx3^tm1ltan^* (*Runx3^fl^*), *Bax^−/−^*, *TrkC^−/−^* and Ai14 mice were purchased from Jackson Laboratories, and *NT3^−/−^* from MMRRC. Both male and female animals were included in this study, except for the *Adv^Cre^*; *Runx3^fl/fl^*, where only males were analyzed because of weak expression of Cre in oocytes and possible leakage or generation of ‘deletor’ mice. Animals were group-housed, with food and water available *ad libitum*, under 12 h light-dark cycle conditions. Fertile white Leghorn eggs were incubated at 38°C and embryos were staged according to Hamburger-Hamilton (HH) tables. All animal work was performed in accordance with the national guidelines and approved by the local ethics committee of Stockholm, Stockholms Norra djurförsöksetiska nämnd.

### *In vitro* cultures of whole DRG

Whole DRG were cultured on coated plates (5% matrigel in ice-cold PBS) with N2 medium (DMEM-F12/glutamax medium with N2 supplement; Gibco) supplemented with pen/strep, gentamicin, the pan-caspase inhibitor Q-VD-Oph (2 μM, Sigma) and with NT3 (Peprotech), retinoic acid, bFGF and IGF when specified, as previously described (Hadjab et al., 2013). DRG analyzed for mRNA expression were cultured directly in eppendorf tubes.

### qPCR

Tissue was freshly dissected and directly placed into lysis buffer. RNA was extracted using the Qiagen RNAeasy Mini Kit according to the manufacturer's instructions, including DNase I to degrade potential genomic DNA contamination. The amount of RNA was quantified using the Qubit RNA BR Assay Kit, and 50 ng of each biological replicate used for reverse transcription using Biorad iScript in a 20 µl reaction according to the manufacturer's instructions. Produced cDNAs were used to analyze transcript levels using real-time PCR in a Biorad CFX96 Real-time System and using Biorad iTaq Universal SYBR Green Supermix in 20 µl reactions for 40 cycles (95°C for 10 s, 60°C for 20 s, 72°C for 30 s followed by fluorescence measurement at 75°C to reduce the influence of primer dimer on quantification). Primers for *Nft3* and *Runx3* were designed using Primer3 Blast (NCBI). Primers for *Nft3*, *Ntrk3* (TRKC), *Isl1* and *Runx3* were designed using Primer3 Blast (NCBI). *Gapdh* was used as a reference gene. A full list of primers used is given below. Negative controls did not amplify. Data were analyzed using Bio-Rad CFX manager. Gapdh, AACTCCCACTCTTCCACCTTC and GATAGGGCCTCTCTTGCTCAG; Nft3, ATAAAATTCGTGTGCTTGCCTTCC and GAGAGCCCAATCACAAAACAAGG; Runx3, ACCAAGTGGCGAGATTTAACGA and ACGGTGACTTTAATGGCTCGG; Isl1, AAAAGAAGCATTATGATGAAGCAA and CATGTCTCTCCGGACTAGCAG; Ntrk3, TGATCCTCGTGGATGGGCAG and CTTCACCAGCAGGTTGGCTCC.

### Immunostaining

Mouse embryos were collected and fixed for 1 to 6 h at +4°C (4% PFA in PBS) depending on the stage, washed in PBS three times (30 min each), equilibrated in 20% and 30% sucrose in PBS, embedded in OCT (Tissue-Tek) and cryosectioned at 14 μm. Sections were incubated for one or two nights with primary antibodies diluted in blocking solution (2% donkey serum, 0.0125% NaN_3,_ 0.5% Triton X-100 in PBS). Primary antibodies used were: rabbit anti-RUNX3 (a gift from T. M. Jessell and D. Levanon, Columbia University and the Weizmann Institute of Science), goat anti-TRKA (R&D Systems, AF1056, 1/500), goat anti-TRKB (R&D Systems, AF1494, 1/500), goat anti-TRKC (R&D Systems, AF1404, 1/500), goat anti-RET (R&D Systems, AF482, 1/500), mouse anti-ISL1 (Developmental Studies Hybridoma Bank, 39.4D5, 1/200), rabbit anti-VGLUT1 (SYSY 135303, 1/1000), chicken anti-RFP (Rockland, 600-401-379, 1/500), goat anti-ChAT (Millipore, AB144p, 1/1000), mouse anti-NF200 (Sigma, N0142), rabbit anti-peripherin (Millipore, AB1530, 1/500), rabbit anti-PEA3 (a gift from S. Arber, Friedrich Miescher Institute, Basel, Switzerland) and mouse anti-myosin (DSHB, F59-s, 1/50). After washing with PBS, Alexa Fluor secondary antibodies (Life Technology; 1:500 in blocking solution) were applied overnight (at +4°C). Samples were then washed in PBS and mounted in DAKO fluorescent mounting medium. Staining was imaged by confocal microscopy (Zeiss LSM700 or LSM800) using identical settings between control and experimental groups. Optical pinholes were 2 μm under 20× magnification unless specified otherwise.

### Quantification of neurons

For cell type counting quantifications, ImageJ software was used. Only neurons with a visible nucleus were considered for analysis. Quantification of molecular markers in the DRG was carried out on five DRG sections/animal, selected from the most equatorial region of each DRG and covering the segments C5-T1 (see figure legends for *n* values and genotypes).

### Quantification of muscle spindles

Limb muscle VGLUT1^+^ muscle spindles were counted on longitudinal sections of whole muscles.

### Quantification of PSN collateral density

Quantitative analysis of PSN axon terminals in the ventrolateral horn of the spinal cord was performed using ImageJ analysis software. For each segment level (C5 and C8) the total VGLUT1^+^ collateral surface area (expressed as squared microns) was measured within a confined intermediate zone, ventromedial area and ventrolateral area of the ventral horn (adjusted for each segmental level). The surface areas analyzed were as follows: C5 ventrolateral, 210,000 µm^2^; C5 ventromedial, 112,000 µm^2^; C5 intermediate zone, 37,400 µm^2^; C8 ventrolateral, 160,000 µm^2^; C8 ventromedial, 63,000 µm^2^; and C8 intermediate zone, 30,000 µm^2^. For each genotype, measurements for all segmental levels were performed from at least two individual experiments and three *z*-stacks per segment.

### Quantification of sensory synaptic contacts with motor neurons

Quantification of VGLUT1^+^ sensory bouton contacts between PSNs and motor neurons somata was performed using 0.5 µm confocal *Z*-scans of 10 to 20 µm thick sections. Motor neuron surface area was determined using Image J. Synapses were counted manually and filtered for sizes larger than 1 µm^2^.

### β-Galactosidase staining

*LacZ* expression was detected by staining using X-Gal (5-bromo-4-chloro-3-indolyl β-d-galactoside) for β-galactosidase activity as described previously (Lallemend et al., 2012).

### Retrograde labeling of motor and sensory neurons

Newborn mice were anesthetized on ice by hypothermia and muscles of interest were injected with 1% cholera toxin B subunit/Alexa555 (CTB555). The amount of CTB555 was enough to fill the whole muscle. The following day, spinal columns were dissected and fixed in 4% PFA for 6 h then processed for immunostaining and analysis. Sensory neuron tracing was performed *ex vivo* by injection of fluorescently labeled dextrans (3000MW, Invitrogen) in limb muscles using tightly fitting glass capillaries. Following injection, the *ex vivo* preparation (brachial region with associated limbs) was incubated for 8 h in oxygenated ACSF before fixation.

### Statistics

Data were analyzed using GraphPad Prism 6 and are expressed as mean±s.e.m. The statistical test performed is reported in the figure legend. *t*-tests were two-sided (*P≤0.05, **P≤0.01, ***P≤0.001). No animals or data points were excluded from the analyses. No statistical methods were used to pre-determine sample size but our sample sizes are similar to those generally employed in the field.

## Supplementary Material

Supplementary information
