# Supplementary material for: Muscle-selective RUNX3 dependence of sensorimotor circuit development
Source: Development. 2019 Oct 24;146(20):dev181750. doi: 10.1242/dev.181750 (PMC6826036; doi:10.1242/dev.181750)
Supplement: Supplementary information [file develop-146-181750-s1.pdf]

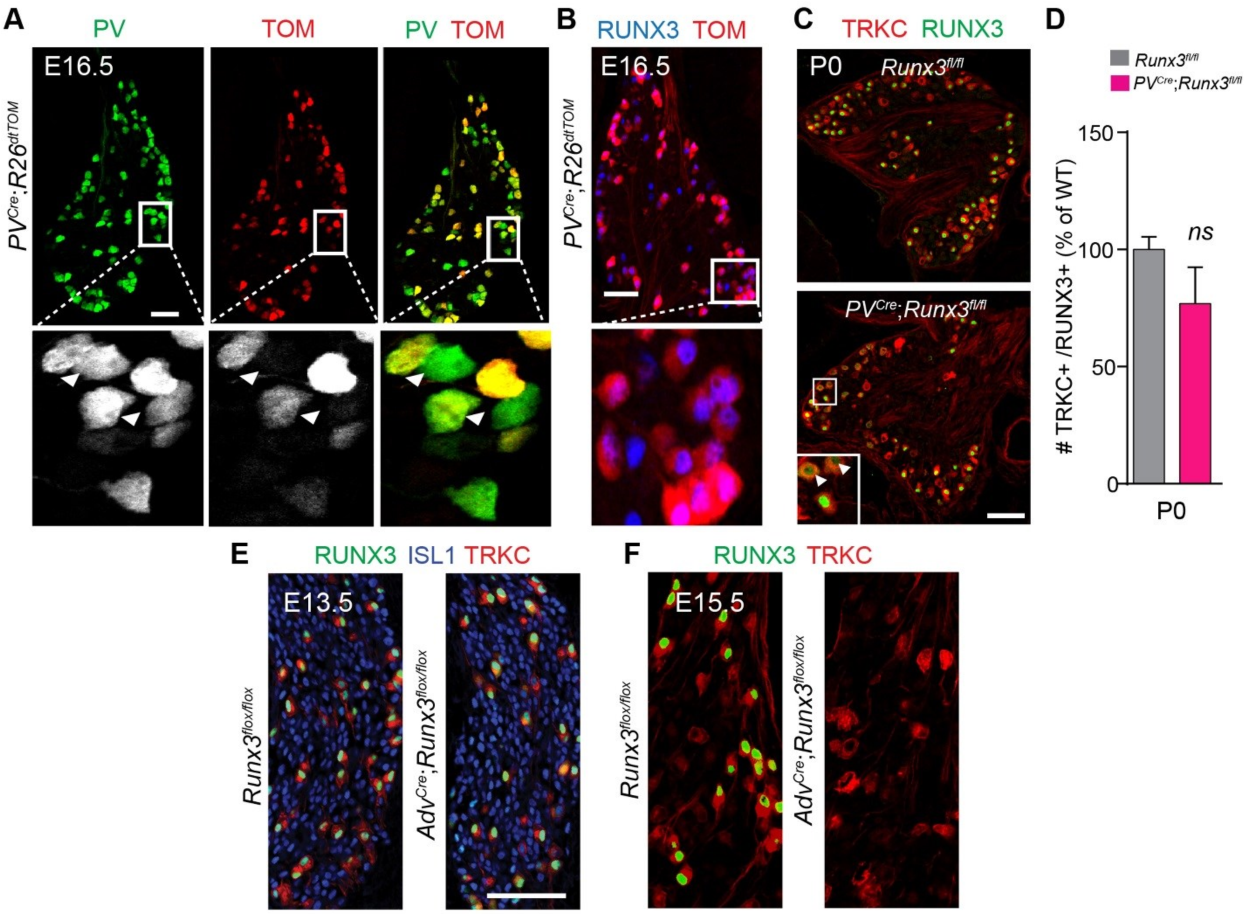

Fig. S1. related to Figure 1. Conditional targeting of RUNX3 in sensory neurons. (A) Immunostaining for PV and TOM expression on DRG section from E16.5 *PV^Cre^;R26^tdTOM^* animals. Scale bar: 100µm. (B) RUNX3 expression is not affected in *PV^Cre^;Runx3^fl/fl^* at E16.5. Scale bar: 100µm. (C,D) RUNX3 is still expressed in ~80% of the PSNs at P0 in the *PV^Cre^;Runx3^fl/fl^* mice. Arrowheads in (C) indicate lower RUNX3 expression in few PSNs. Scale bar: 100 µm. (E) Immunostaining for ISL1, RUNX3 and TRKC in DRG sections of E13.5 *Adv^Cre^;Runx3^fl/fl^* and *Runx3^fl/fl^* embryos shows that RUNX3$^+$ cell number remain unchanged (quantification in Figure 1C). Scale bar: 200µm. (F) Immunostaining for RUNX3 and TRKC in DRG section of E15.5 *Adv^Cre^;Runx3^fl/fl^* and *Runx3^fl/fl^* embryos show an almost complete loss of RUNX3$^+$ cell in the full mutants (quantification in Figure 1C). Scale bar: 200µm.

Development • Supplementary information

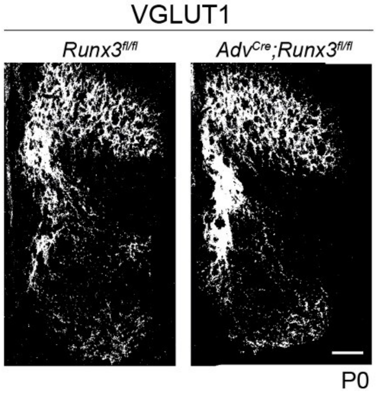

Fig. S2. related to Figure 2. Conditional targeting of RUNX3 in PSN does not affect the central afferentation at the thoracic level. (A) Central afferentation of PSNs in *Adv*^{Cre};*Runx3*$^{fl/fl}$ and *Runx3*$^{fl/fl}$ mice at thoracic levels as revealed by VGLUT1 immunostaining. Quantification of the density of VGLUT1 staining in regions merging intermediate zone (IZ) and the ventromedial (M) region on one side of the spinal cord (as described in Fig. 2A) reveals similar central ingrowth of PSNs afferents in conditional *Runx3* mutant mice compared to Ctr mice; *Runx3*$^{fl/fl}$: 100; *Adv*^{Cre};*Runx3*$^{fl/fl}$: 111%; % of *Runx3*$^{fl/fl}$, showing average from 6 sections of the thoracic region (T4-T10); similar observation has been done in 3 animals. Scale bar: 100µm.

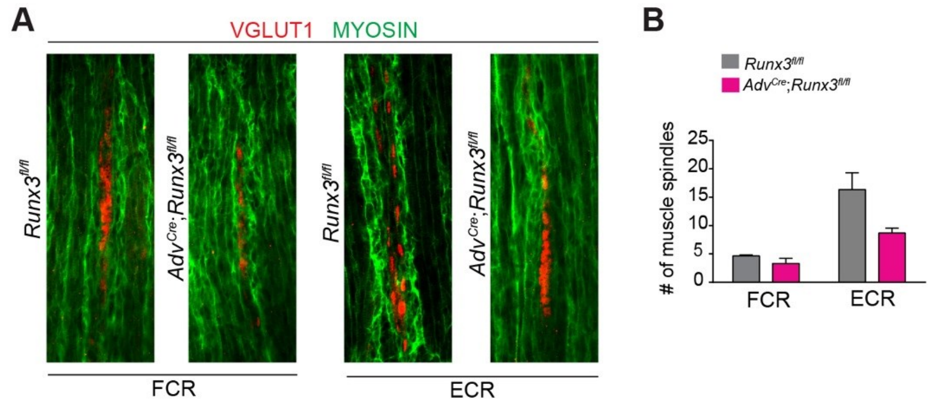

Fig. S3. related to Figure 4. Muscle-specific MSs deficits in conditional *Runx3* mutants at birth. (A,B) Immunostaining for VGLUT1 (labelling MSs) and MYOSIN on cross-sections from FCR and ECR muscles. Quantification in (B) reveals a muscle-selective MS deficiency in Adv^{Cre};$Runx3^{fl/fl}$ with a ~40% decrease in the number of MSs in ECR while the MSs in FCR remained unchanged. N=3 animals, *ns,* non-significant, **$P \leq 0.001$; Student's t test. Data are presented as mean ± SEM. Scale bar: 100µm.

Development • Supplementary information

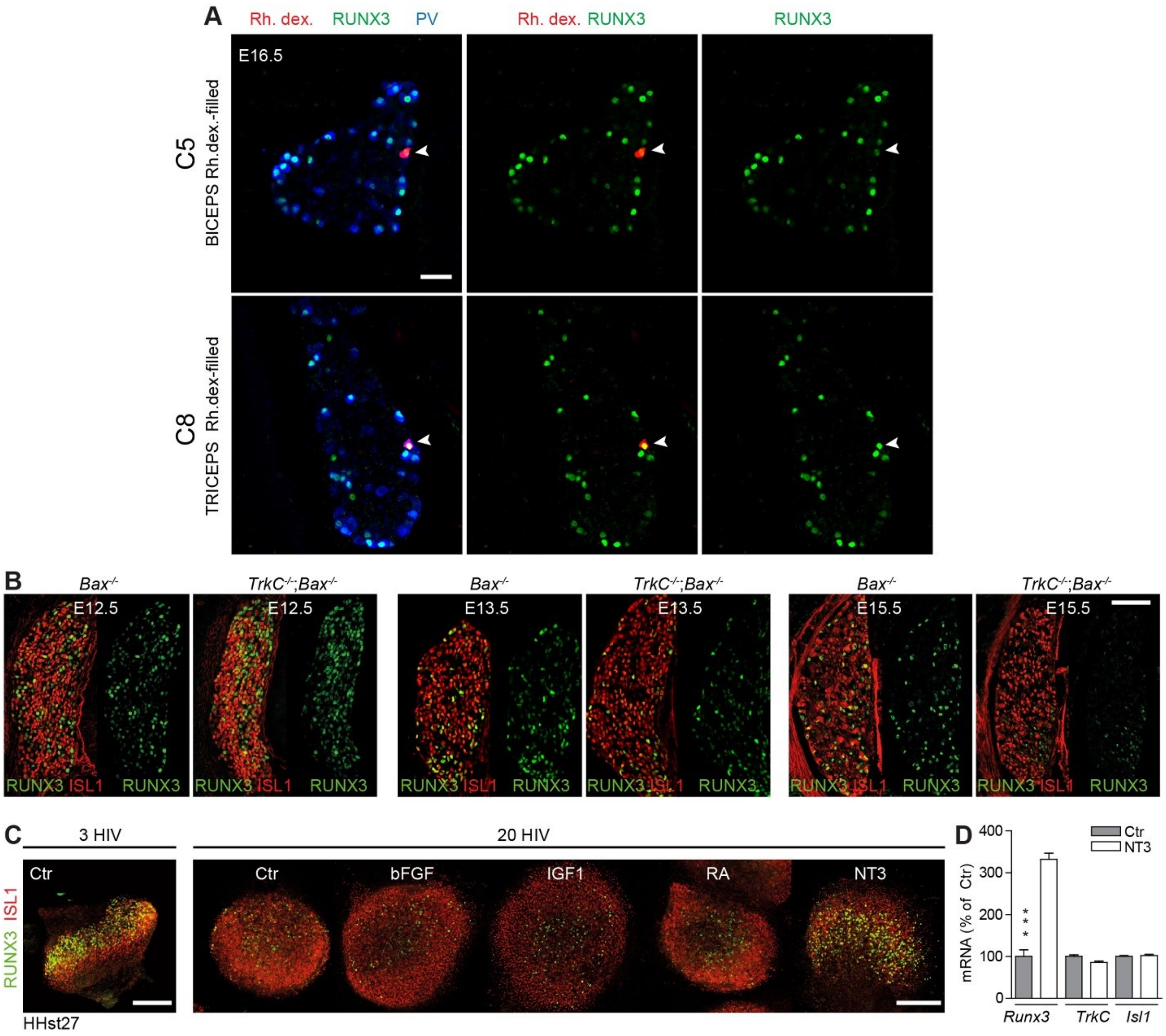

Fig. S4. related to Figure 5. RUNX3 regulation by NT3-TRKC signaling. (A) C5 and C8 DRG sections from E16.5 embryos showing backfilled traced PSNs following injection of Rhodamine dextran in either biceps (C5, Rh.dex., top panels) or triceps (C8, Rh.dex., lower panels). Sections were immunostained for RUNX3 and PV, revealing that biceps-innervating PSNs (Rh.dex+) express low levels of RUNX3 compared to all PSNs, while triceps-innervating PSNs (Rh.dex+) express high levels of RUNX3. See quantification in Fig. 1D. Scale bar: 50µm. (B) RUNX3 expression is progressively reduced in *TrkC*$^{-/-}$;*Bax*$^{-/-}$ mice, a mouse model showing peripheral outgrowth deficits. Cross-sections of DRG are immunostained for ISL1 and RUNX3 at E12.5, E13.5 and E15.5. RUNX3 expression is not affected at E13.5 and largely reduced by E15.5 (n=3). Scale bar: 100µm. (C) Whole chicken DRG cultures. Brachial DRG were dissected out at HHst27 and cultured for 20 hours (20HIV) with different factors, NT3, retinoid acid (RA), IGF and bFGF. Of all tested factors, only NT3 supplemented medium led to the maintenance of RUNX3 expression. Scale bar: 100µm. (D) Quantification of *Runx3*, *TrkC* and *Isl1* mRNA expression in NT3 *versus* Control conditions by qPCR. Thoracic DRG from HHst27 were cultured for 6 hours. ***$P \leq 0.001$; Student's t test. Data are presented as mean ± SEM.

Development • Supplementary information
